# 61 Allogenic Plasma Unit Reference Ranges for Select Biomarkers and Thrombin and Lysis Dynamics

**DOI:** 10.1093/jbcr/iraf019.061

**Published:** 2025-04-01

**Authors:** Meghan Fondakowski, Quinn Chapman, Samuel DiPasquale, Thomas Orfeo, Matthew Gissel, Melissa McLawhorn, Anthony Pusateri, Lauren Moffatt, Jeffrey Shupp, Maria Cristina Bravo

**Affiliations:** University of Vermont; Case Western Reserve University; University of Vermont; University of Vermont; University of Vermont; MedStar Health Research Institute; Naval Medical Research Unit San Antonio; MedStar Health Research Institute; MedStar Washington Hospital Center; University of Vermont

## Abstract

**Introduction:**

Burn patients may be transfused with healthy donor plasma as a part of plasma inclusive resuscitation as part of the treatment of burn shock. Here we performed a comprehensive assessment of fresh frozen plasma (FFP) transfusion units that were subsequently used at a single regional burn center.

**Methods:**

Aliquots of FFP units (n=29) that were administered to burn injured patients were collected and frozen. Thrombin generation potential was assessed using the calibrated automated thrombogram protocol. Reactions were conducted in the presence of exogenously added tissue factor (TF) ± thrombomodulin (TM) to assess the contribution of the protein C pathway. Thrombin generation was described by parameters of lag time, peak thrombin, and endogenous thrombin potential. Fibrinolytic potential was assessed using a clot lysis assay where plasma samples were incubated with exogenous TF ± tissue-type plasminogen activator (tPA). Clot formation and lysis dynamics were measured optically, and parameters of clot formation, lysis onset, and lysis completion were extracted. A reference healthy donor plasma pool was run alongside thrombin generation and clot lysis assays. Activity levels of FVII, FVIII, and PAI-1 were measured. Data are presented as mean ± SD.

**Results:**

Peak thrombin levels in transfusion units were 104.4 ± 30.5 nM and 119.5 ± 14.7 nM thrombin in reference plasma. Units had a robust response to the presence of TM with an 80% ± 12% reduction in peak thrombin generated while our reference plasma reduced by 32% ± 13%. Transfusion units appeared to have a faster lysis onset time in response to tPA compared to our reference control plasma (411 ± 179 s vs. 739 ± 165 s, p < 0.005), however the complete lysis time was not significantly different (1116 ± 750 s vs. 1068 ± 104 s, p = 0.7). FVII and FVIII levels were within the accepted normal healthy range (81% ± 25% and 91% ± 33%, respectively, of mean physiological levels). We measured active PAI-1 at 3.8 ± 5.05 U/mL, within reported healthy normal ranges.

**Conclusions:**

FFP units do not present with an abnormal profile of fibrinolytic potential or select biomarkers of coagulation. The robust response of FFP units to TM in TF-initiated thrombin generation assays suggests that administration of these FFP units would not result in a significant source of added procoagulant potential.

**Applicability of Research to Practice:**

FFP administration would not be expected to result in an abnormal contribution to coagulation or fibrinolytic potential.

**Funding for the Study:**

USAMRAAA - BA190086

